# Prognostic relevance of the hexosamine biosynthesis pathway activation in leiomyosarcoma

**DOI:** 10.1038/s41525-021-00193-w

**Published:** 2021-05-03

**Authors:** Angela Tolwani, Magdalena Matusiak, Nam Bui, Erna Forgó, Sushama Varma, Lucia Baratto, Andrei Iagaru, Alexander J. Lazar, Matt van de Rijn, Joanna Przybyl

**Affiliations:** 1 Department of Pathology, Stanford University School of Medicine, Stanford, CA USA; 2 Department of Medicine, Stanford University School of Medicine, Stanford, CA USA; 3 Department of Radiology, Division of Nuclear Medicine, Stanford University Medical Center, Stanford, CA USA; 4 Departments of Pathology and Translational Molecular Pathology, The University of Texas MD Anderson Cancer Center, Houston, TX USA

**Keywords:** Sarcoma, Prognostic markers, Cancer metabolism, Gene regulatory networks, Data mining

## Abstract

Metabolic reprogramming of tumor cells and the increase of glucose uptake is one of the hallmarks of cancer. In order to identify metabolic pathways activated in leiomyosarcoma (LMS), we analyzed transcriptomic profiles of distinct subtypes of LMS in several datasets. Primary, recurrent and metastatic tumors in the subtype 2 of LMS showed consistent enrichment of genes involved in hexosamine biosynthesis pathway (HBP). We demonstrated that glutamine-fructose-6-phosphate transaminase 2 (GFPT2), the rate-limiting enzyme in HBP, is expressed on protein level in a subset of LMS and the expression of this enzyme is frequently retained in patient-matched primary and metastatic tumors. In a new independent cohort of 327 patients, we showed that GFPT2 is associated with poor outcome of uterine LMS but not extra-uterine LMS. Based on the analysis of a small group of patients studied by ^18^F-FDG-PET imaging, we propose that strong expression of GFPT2 in primary LMS may be associated with high metabolic activity. Our data suggest that HBP is a potential new therapeutic target in one of the subtypes of LMS.

## Introduction

Cancer cells have altered metabolic pathways that support the enhanced proliferation and growth^1^. Glucose metabolism generates intermediates that can supply glycolysis and subsidiary pathways including the hexosamine biosynthesis pathway (HBP), the pentose phosphate pathway, and one-carbon metabolism, all of which promote cancer cell growth^2^. Increased consumption of glucose in tumors can be evaluated by positron emission tomography (PET) imaging of the uptake of a radioactive fluorine-labeled analog of glucose, ^18^F-fluorodeoxyglucose (^18^F-FDG). This technology is routinely used in the clinic for tumor diagnosis, staging, and evaluation of response to treatment^1^.

Leiomyosarcoma (LMS) is a highly malignant tumor type associated with poor prognosis^3^. LMS is defined by its smooth muscle differentiation and accounts for 10–20% of all soft tissue sarcomas^3,4^. Several gene expression studies have shown that LMS tumors can be classified into three distinct transcriptomic subtypes^5–8^. We previously described that subtype 1 of LMS is associated with a muscle-enriched gene expression profile, subtype 2 shows an association with undifferentiated pleomorphic sarcoma, and subtype 3 is strongly enriched in uterine cases^6^. In the present study, we sought to explore the possible differences in metabolic reprogramming between these three molecular subtypes of LMS. We found a remarkable enrichment of genes involved in the HBP in subtype 2 of LMS and focused on the expression pattern and prognostic significance of glutamine-fructose-6-phosphate transaminase 2 (GFPT2), the first and rate-limiting enzyme in HBP. Activation of HBP leads to altered O-GlcNAcylation and N-/O-glycosylation of transcription factors and kinases in many types of cancer^9,10^. These aberrations may lead to increased proliferation, invasion, metastasis, and survival of tumor cells, and may be associated with resistance to therapy^11,12^. Several studies have demonstrated that HBP is activated in a number of cancers and that targeting of GFPT1/2 can provide therapeutic benefit^13^. In addition, GFPT2 has been associated with poor prognosis and high glucose uptake in cells of mesenchymal origin, i.e., in cancer-associated fibroblasts in lung adenocarcinoma^14^, but the activation of GFPT2 has not been investigated in mesenchymal tumors.

## Results

### Consistent enrichment of amino sugar and nucleotide sugar metabolism genes in primary, recurrent, and metastatic LMS

In order to explore the possible metabolic reprogramming in LMS, we interrogated transcriptomic profiles of tumors analyzed in four independent studies published by Guo et al., Beck et al., Chudasama et al., and by TCGA^5–8^. All of these studies classified LMS tumors into three distinct subtypes based on their gene expression profiles. In the present study, we performed Gene Set Enrichment Analysis (GSEA) in distinct LMS subtypes defined in these four datasets with the goal of identifying possible differences in expression levels of 14 curated carbohydrate metabolism gene sets that are cataloged in Kyoto Encyclopedia of Genes and Genomes (KEGG)^15,16^.

**Fig. 1:**
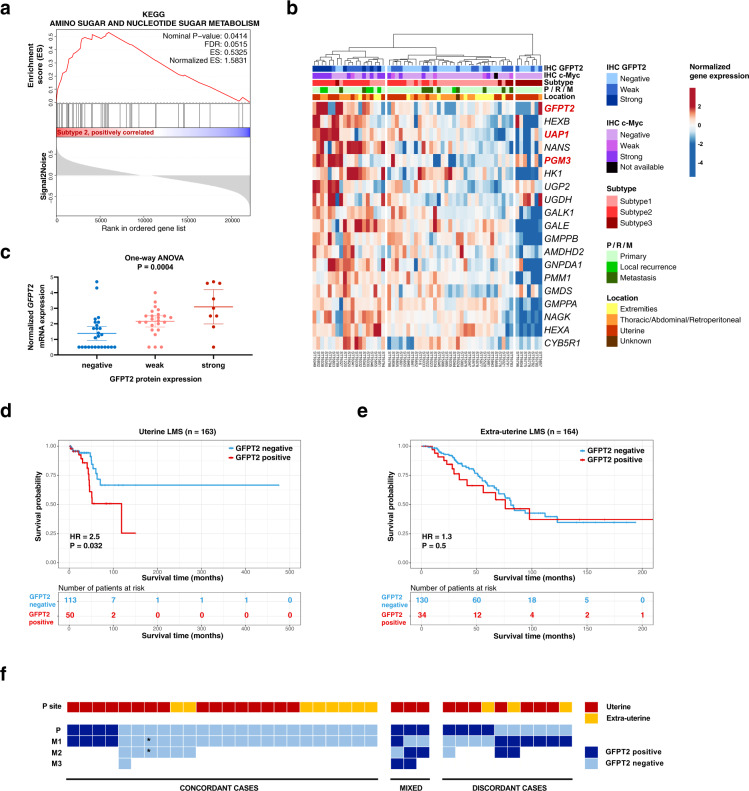
Expression of hexosamine biosynthesis pathway genes and prognostic significance of GFPT2 in LMS. **a** Enrichment plot for the KEGG Amino sugar and nucleotide sugar metabolism gene set in subtype 2 of LMS. Profile of the running Enrichment Score and positions of gene set members on the rank ordered list. **b** Supervised clustering of 59 LMS based on expression levels of the 19 genes involved in the amino sugar and nucleotide sugar metabolism that are significantly overexpressed in subtype 2 of LMS compared to subtypes 1 and 3. Genes in red font encode three enzymes of hexosamine biosynthesis pathway. Gene expression values were normalized using variance stabilizing transformation, followed by row centering and row scaling. GFPT2 and c-Myc protein expression, transcriptomic subtype, tumor type and location of the primary tumor are indicated for each specimen in the top panel (IHC immunohistochemistry, P primary tumor, R local recurrence, M distant metastasis). **c** Correlation between the levels of *GFPT2* mRNA and protein expression in 59 LMS tumors. Gene expression was normalized using variance stabilizing transformation (the plot represents median normalized gene expression values with 95% confidence intervals). **d**, **e** Correlation between the overall survival and GFPT2 protein expression determined by immunohistochemistry in patients with uterine and extra-uterine LMS (HR hazard ratio). Tumors with weak or strong expression of GFPT2 were classified as “GFPT2 positive” (red line) as opposed to “GFPT2 negative” tumors (blue line). Censored observations are indicated on the Kaplan–Meier curves with a cross. **f** Consistency of GFPT2 protein expression or lack of expression between primary and metastatic tumors in 37 patients (P primary tumor; M1, M2, M3 metastatic tumors; asterisk indicates two metastatic tumors removed at the same time).

First, we re-analyzed 70 LMS tumors previously profiled by 3′-end RNA sequencing by our group (GSE45510)^6^. This cohort included 52 primary tumors and 18 recurrent and metastatic tumors that were assigned into three molecular subtypes (Table 1)^6^. We performed GSEA on 35 tumors classified into subtype 1 of LMS, 22 tumors classified into subtype 2, and 13 tumors classified into subtype 3. The two most significantly enriched pathways were tricarboxylic acid (TCA) cycle and amino sugar and nucleotide sugar metabolism in subtype 2 of LMS (false discovery rate (FDR) equal 0.003 and 0.05, respectively) (Table 2) (Fig. 1a). We also performed GSEA separately for primary and recurrent/metastatic tumors in each subtype, and we found that both primary and recurrent/metastatic tumors belonging to subtype 2 showed a consistent enrichment of genes involved in these same two pathways, i.e., TCA cycle and the amino sugar and nucleotide sugar metabolism (Supplementary Table 1). Subtype 1 of LMS in this dataset showed enrichment of genes involved in the inositol phosphate metabolism in primary tumors (FDR = 0.1) but not in recurrent/metastatic tumors (Supplementary Table 1). In primary tumors classified as subtype 3, we found enrichment of genes involved in ascorbate and aldarate metabolism and in pentose and glucuronate interconversions (FDR equal 0.05 and 0.23, respectively), but the number of recurrent/metastatic tumors from subtype 3 was insufficient to perform analogical analysis (Supplementary Table 1).

**Table 1:** Overview of patient demographics.

	Stanford LMS subtypes cohort	Survival cohort	Imaging cohort
Number of patients	70	327	16
Median age at diagnosis (range)	56 (2–84)	52 (22–84)	63 (28–82)
Gender			
Female	52	271	11
Male	18	53	5
Unknown		3	
Location of primary tumor			
Uterine	34	163	3
Extra-uterine	35	164	8
Unknown	1		5
Grade			
High	40	71	NA
Intermediate	14	29	NA
Low	16	3	NA
Unknown		224	

*NA* not available.

**Table 2:** Gene Set Enrichment Analysis of KEGG metabolic pathways in 70 LMS classified into three molecular subtypes.

	Gene set name	ES	NES	FDR	Number of genes in core enrichment
Subtype 1	KEGG Inositol phosphate metabolism	0.49	1.56	0.22	28/54
Subtype 2	KEGG Citrate cycle, TCA cycle	0.65	1.84	0.003	16/30
Subtype 2	KEGG Amino sugar and nucleotide sugar metabolism	0.53	1.58	0.05	28/43
Subtype 2	KEGG Glyoxylate and dicarboxylate metabolism	0.50	1.33	0.23	7/16
Subtype 3	KEGG Ascorbate and aldarate metabolism	0.49	1.379	0.16	15/25

*ES* enrichment score, *NES* normalized enrichment score, *FDR* false discovery rate.

Next, we performed GSEA in the gene expression datasets published by Beck et al., Chudasama et al., and TCGA. We found that amino sugar and nucleotide sugar metabolism was significantly enriched in the cases assigned to subtype 2 in the study of Chudasama et al. (FDR = 0.19) (Supplementary Table 2). Amino sugar and nucleotide sugar metabolism was also the only gene set identified by GSEA in subtype 2 of LMS described by Beck et al., but this result had a high FDR in this cohort (FDR = 0.5) (Supplementary Table 3). Remarkably, cases classified as subtype 2 of LMS in these three independent datasets published by Guo et al., Chudasama et al., and Beck et al. were demonstrated to have matching molecular profiles defined by overexpression of the ARL4C marker^5–7^. Amino sugar and nucleotide sugar metabolism genes that were significantly enriched in subtype 2 in these independent datasets included the key enzymes of the HBP (Figs. 1b and 3) (Supplementary Tables 4–7). The *GFPT2* gene that encodes the first and rate-limiting enzyme in HBP, was the top enriched gene in the datasets of Chudasama et al., Beck et al., and in the recurrent and metastatic tumors in our dataset of 70 LMS (Supplementary Tables 5–7).

Integrative consensus clustering performed by TCGA on 206 sarcoma specimens, including 80 LMS, defined distinct subclusters of LMS, both in comparison with other sarcomas and within LMS. However, we did not find enrichment of the amino sugar and nucleotide sugar metabolism genes within the LMS subclusters identified by TCGA^8^.

Next, we sought to confirm the overexpression of amino sugar and nucleotide sugar metabolism genes in subtype 2 of LMS by differential expression analysis. We performed multiclass differential expression analysis using SAMseq to identify genes upregulated in subtypes 1, 2, and 3 in our dataset of 70 LMS. We found 3584 genes significantly overexpressed in subtype 2 LMS compared to subtypes 1 and 3 (contrast > 2, FDR < 0.05). Among these genes that were significantly overexpressed in subtype 2 of LMS, we identified 19 genes from the KEGG amino sugar and nucleotide sugar metabolism gene set (Supplementary Table 8) (Fig. 1b). In addition to the genes included in the KEGG amino sugar and nucleotide sugar metabolism gene set, differential expression analysis also showed that subtype 2 of LMS significantly overexpresses *OGT*, a gene encoding glycosyltransferase that catalyzes the addition of the O-GlcNAc in posttranslational modification of proteins as a result of HBP activation (Fig. 3).

### Validation of GFPT2 protein expression in subtype 2 of LMS

**Fig. 2:**
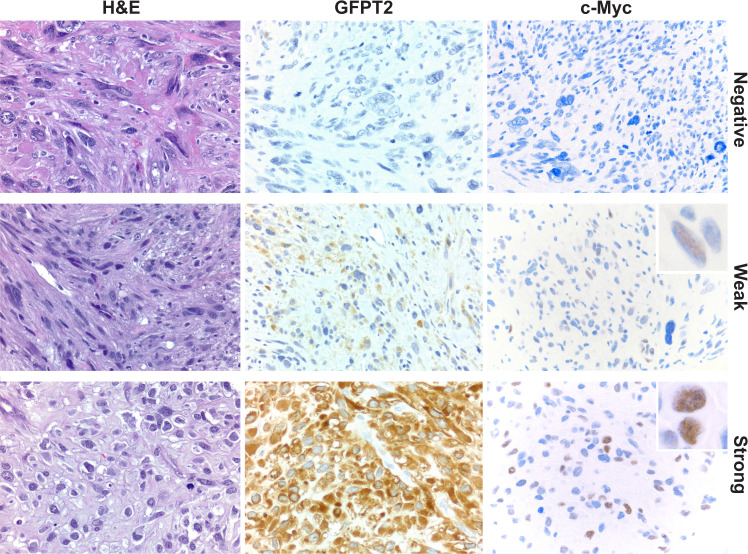
Histologic appearance of three high grade LMS cases with different patterns of cytoplasmic expression of GFPT2 and nuclear expression of c-Myc. The first row shows representative H&E and immunohistochemical staining in LMS that lacks GFPT2 and c-Myc expression. The second row shows LMS with weak cytoplasmic expression of GFPT2 and a concordant weak nuclear expression of c-Myc in ~5% of cells. The third row demonstrates LMS with strong cytoplasmic expression of GFPT2 and a concordant strong nuclear expression of c-Myc in ~30% of cells. Total magnification ×600. (H&E hematoxylin and eosin).

GFPT2 is the first and rate-limiting enzyme in HBP and it was the top enriched gene in this pathway in our recurrent and metastatic specimens, and in the datasets of Chudasama et al. and Beck et al., thus we further focused on its possible role in LMS. The GFPT2 protein has a relatively short half-life of less than an hour and it has been proposed that its levels appear to be regulated mainly by gene expression^17^. In order to validate overexpression of GFPT2 on the protein level in one of the molecular subtypes of LMS, we performed immunohistochemistry (IHC) with a monoclonal antibody against GFPT2 on 59 of the 70 tumor specimens that had been used for the transcriptomic analysis (Fig. 1b). LMS tumors were scored as strongly positive, weakly positive, or negative for cytoplasmic expression of GFPT2 and representative stains are shown in Fig. 2. LMS tumors that expressed GFPT2 protein had higher expression levels of *GFPT2* transcript compared to the tumors that did not express GFPT2 protein [one-way ANOVA test *p* value = 0.0004, *F*(2, 56) = 8.95] (Fig. 1c). GFPT2 protein was expressed in the majority of subtype 2 LMS cases (86%, 19/22) compared to 35% (9/26) and 45% (5/11) cases of subtypes 1 and 3, respectively (Fig. 1b) (Table 3). The majority of the cases with strong expression of GFPT2 were classified into subtype 2 of LMS (7/9 cases) (Fig. 1b). These observations confirm that LMS with elevated *GFPT2* mRNA levels showed increased levels of this enzyme also on protein level, and that the expression of GFPT2 protein is enriched in subtype 2 of LMS.

**Table 3:** Expression of GFPT2 and c-Myc proteins in transcriptomic subtypes of LMS.

	Subtype 1 LMS	Subtype 2 LMS	Subtype 3 LMS	Two-sided Chi-square test *p* value
GFPT2 (+)	9	19	5	0.001
GFPT2 (−)	17	3	6	
c-Myc (+)	5	13	1	0.003
c-Myc (−)	20	9	10

**Fig. 3:**
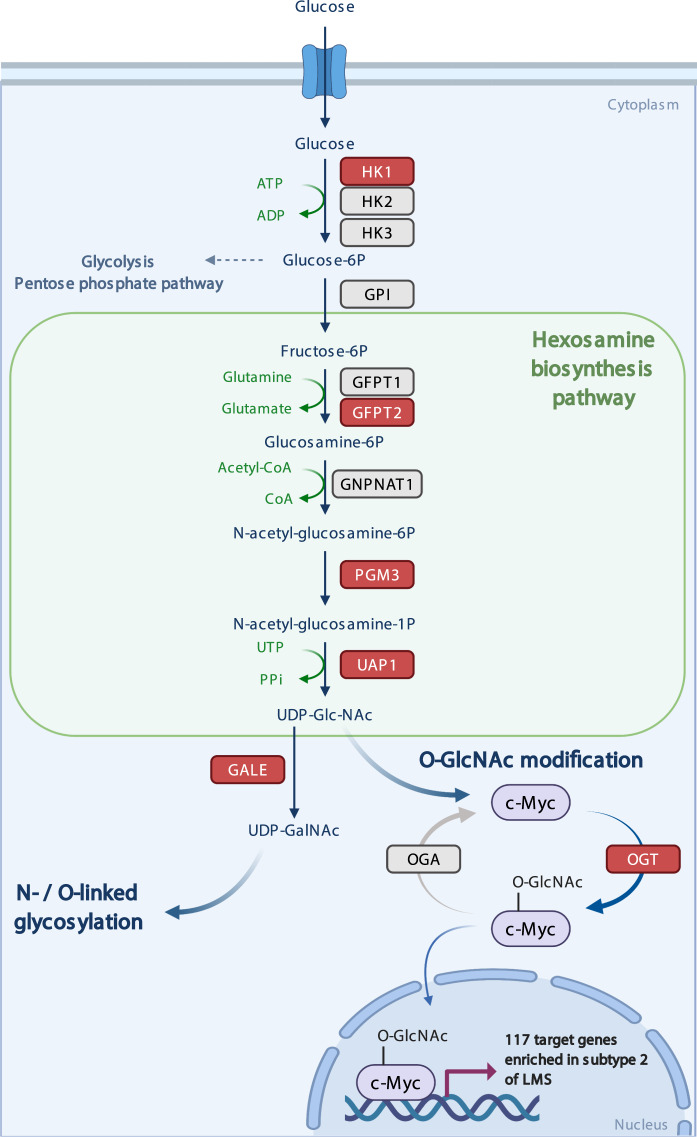
Proposed model of the hexosamine biosynthesis pathway activation in subtype 2 of LMS. Enzymes encoded by the genes that are significantly overexpressed in subtype 2 of LMS are highlighted in red. Figure created with BioRender.com. (HK1/2/3 hexokinases 1/2/3, GPI glucose 6-phosphate isomerase, GFPT1/2 glutamine:fructose-6-phosphate transaminase 1 and 2, GNPNAT1 glucosamine-6-phosphate N-acetyltransferase, PGM3 phosphoacetylglucosamine mutase, UAP1 UDP-N-acetylhexosamine pyrophosphorylase 1, GALE UDP-N-acetylgalactosyl (or UDP-galactosyl)-4 epimerase, OGT O-GlcNAc transferase).

### GFPT2 expression is associated with poor outcome in uterine LMS

We evaluated the prognostic relevance of GFPT2 protein expression in the primary untreated tumors in a separate cohort of 327 patients with localized disease at the time of diagnosis (163 uterine and 164 extra-uterine LMS, with a median follow up time of 34 months, range: 1–475 months). In this cohort, 26% (84/327) of tumors expressed GFPT2 (showing either weak or strong expression). GFPT2 expression was predictive of poor overall survival in patients with uterine LMS (hazard ratio = 2.5, 95% CI = 1.1–5.8, Mantel–Cox log-rank test *p* value = 0.03) but was not associated with clinical outcome in extra-uterine LMS (hazard ratio = 1.3, 95% CI = 0.65–2.4, Mantel–Cox log-rank test *p* value = 0.5) (Fig. 1d, e). Among the uterine tumors, 113 were negative and 50 were positive for GFPT2 protein expression. In the extra-uterine cases, 130 were negative and 34 were positive for GFPT2 protein expression (Fig. 1d, e).

We sought to validate these results in the independent cohort of 80 patients with *GFPT2* mRNA levels measured by RNA-seq in primary LMS included in the TCGA dataset (27 uterine and 53 extra-uterine LMS, with a median follow up time of 37 months, range: 1–174 months)^8^. We divided patients into two groups with tumors showing low expression of *GFPT2* (16 LMS with GFPT2 RSEM values below 20th percentile) and high expression of *GFPT2* (64 LMS with GFPT2 RSEM values above 20th percentile). Even though the TCGA dataset was composed of over four-fold lower number of specimens compared to our survival cohort, there was a trend for association between high expression of *GFPT2* and worse overall survival in the whole cohort of 80 patients (hazard ratio = 2.5, 95% CI = 1.1–5.8, Mantel–Cox log-rank test *p* value = 0.073 and Gehan–Breslow–Wilcoxon test *p* value = 0.04) and also in the limited number of 27 patients with uterine LMS when analyzed separately from extra-uterine LMS (Mantel–Haenszel hazard ratio = 3.55, 95% CI = 0.87–14.49, Mantel–Cox log-rank test *p* value = 0.078) (Supplementary Fig. 1).

### Consistency of GFPT2 expression in primary tumors and relapse

We showed that HBP genes were enriched in primary, recurrent, and metastatic tumors in a subset of LMS. Next, we sought to evaluate whether this pattern is retained on the protein level in individual patients during disease progression. We used tissue microarrays (TMAs) that contained primary tumor and at least one patient-matched distant metastasis or local recurrence for 37 LMS patients (range: 1–3 metastatic tumors per patient) (Fig. 1f). Almost all of these patient-matched primary and metastatic tumors were excised at different time points. Among the 11 patients that showed GFPT2 expression in the primary tumor, 64% (*n* = 7) demonstrated concordant expression of GFPT2 in at least one metastatic tumor (Fig. 1f). For the 26 patients lacking GFPT2 expression in the primary tumor, 77% (*n* = 20) also did not express GFPT2 in any of the examined metastatic tumors (Fig. 1f). The most frequent location of metastasis was in the lungs and 82% (23/28) of lung metastases showed the same pattern of GFPT2 expression as the primary tumor. These results demonstrate the consistency of GFPT2 protein expression in most of patient-matched primary and metastatic/recurrent tumors.

### GFPT2 expression may be correlated with increased glucose uptake in primary LMS

**Fig. 4:**
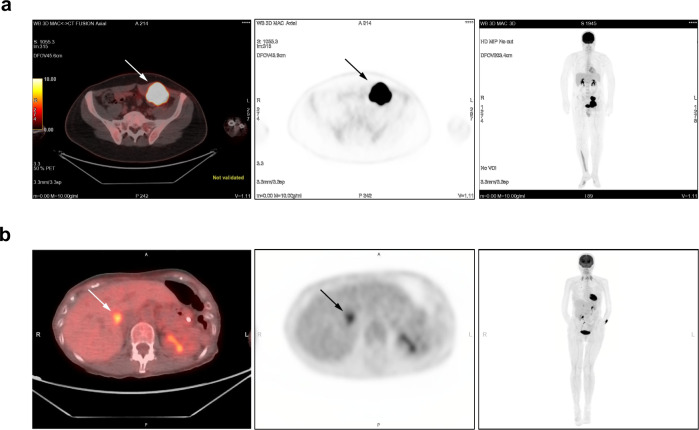
^18^F-FDG-PET images of two LMS patients with tumors showing different patterns of expression of GFPT2. **a** The superior para-rectal mass (5.6 × 7.0 cm) with SUV_max_ of 63 and strong expression of GFPT2. **b** The 3.5 cm mass in vena cava with SUV_max_ of 4.3 and lacking expression of GFPT2. Left panel: fused image, trans-axial view; middle panel: PET image, trans-axial view; right panel: total-body maximum intensity projection image.

Overexpression of GFPT2 was previously associated with increased glucose uptake in cancer-associated fibroblasts in lung adenocarcinoma as evaluated by imaging^14^. We assembled an independent cohort of 16 patients who had undergone ^18^F-FDG-PET imaging (3 patients with primary tumors and 13 patients with metastatic or recurrent tumors) and performed IHC for GFPT2 expression on the same tumors that were analyzed by imaging. Observations from the three primary LMS tumors indicate that GFPT2 expression levels appear to be correlated with glucose uptake (Table 4). A para-rectal LMS tumor with strong expression of GFPT2 protein had a very high glucose consumption expressed as the maximum standardized uptake value (SUV_max_ = 63) (Fig. 4a). The other two primary tumors were negative for GFPT2 protein expression and presented with a low metabolic activity (SUV_max_ of 4.3 and 4.4) (Fig. 4b). In contrast, in metastatic and recurrent LMS tumors we did not observe the association between the expression of GFPT2 protein and elevated SUV_max_. Ten metastatic and recurrent tumors that did not express GFPT2 showed a median SUV_max_ of 12 (range: 2.8–32.6), while three metastases that expressed GFPT2 had a lower median SUV_max_ of 3.3 (range: 2.5–8) (Table 4).

**Table 4:** Maximum standardized uptake value (SUV_max_) and GFPT2 protein expression in 16 LMS patients.

Tumor location	Primary/metastatic/recurrent tumor	Age [years]	Gender	Tumor size [cm]	SUV_max_	GFPT2 protein expression
Rectal muscle	Primary	28	Male	7	63.0 [superior mass^a^]	Positive^a^
				7.4	69.0 [inferior mass]	
Vena cava	Primary	61	Female	6	4.4	Negative
Vena cava	Primary	76	Female	3.5	4.3	Negative
Abdomen	Metastatic	49	Female	13	8.0	Positive
Liver	Metastatic	65	Female	1.3	3.3	Positive
Lung	Metastatic	59	Female	1	2.5	Positive
Liver	Metastatic	69	Female	3	32.6	Negative
Femur	Recurrent	68	Female	4	22.2	Negative
Lung	Metastatic	36	Female	1.2	21.8	Negative
Omentum	Metastatic	54	Female	6	17.0	Negative
Liver	Metastatic	64	Male	2.5	15.5	Negative
Liver	Metastatic	71	Male	14	8.5	Negative
Abdomen	Metastatic	61	Female	15.7	5.9	Negative
Liver	Metastatic	58	Male	NA	5.6	Negative
Gluteus	Metastatic	82	Female	2	3.9	Negative
Vena cava	Recurrent	70	Male	1.7	2.8	Negative

*NA* not available.

^a^Indicates that GFPT2 expression was evaluated in the superior mass.

### Activation of HBP may stabilize c-Myc in subtype 2 of LMS

HBP is responsible for the production of a key substrate for protein glycosylation, UDP-GlcNAc (Fig. 3). Glycosylation may stabilize selected transcription factors in cancer cells and lead to an increased expression of their target genes^18^. Thus, we sought to identify the potential targets of altered glycosylation that may be specific for subtype 2 LMS tumors. We performed GSEA using 570 gene sets curated in MSigDB collection C3 TFT (regulatory target gene sets with all transcription factor targets)^16^. Through this analysis, we identified significant enrichment of target genes regulated by 21 known transcription factors in subtype 2 of LMS (FDR < 0.25) (Supplementary Table 9).

Next, we sought to identify if any of these 21 transcription factors are known to be regulated by glycosylation. We queried two databases with curated posttranslational modifications in human proteins: PhosphoSitePlus^19^ that catalogs known O-GlcNAc sites in 169 human proteins, and the iPTMnet database^20^ that contains curated information of O-glycosylation sites in 159 human proteins and N-glycosylation sites in 1059 in human proteins. Among the 21 transcription factors for which we identified a significant enrichment of their target genes in subtype 2 of LMS, c-Myc is known to be posttranslationally modified by O-GlcNAcylation at threonine 58^19^. By GSEA, we identified significant enrichment of as many as 117/248 genes of the “MYCMAX_03” gene set in subtype 2 of LMS (FDR = 0.23, Supplementary Tables 9 and 10), which suggests the activation of c-Myc oncoprotein in this subtype of LMS. We hypothesize that this may be an effect of c-Myc stabilization by O-GlcNAc modification, sustained by the activated HBP in these tumors (Fig. 3).

In order to validate expression of c-Myc on the protein level in subtype 2 of LMS, we performed IHC with a monoclonal antibody against c-Myc on 58 of the 59 tumor specimens that had been used for the transcriptomic analysis and GFPT2 protein expression analysis (Fig. 1b). LMS tumors were scored as strongly positive, weakly positive or negative for nuclear expression of c-Myc and representative stains are shown in Fig. 2. Overall, strong or weak expression of c-Myc was observed in 17/33 tumors that expressed GFPT2 protein and only in 2/25 tumors that did not express GFPT2 protein (Table 5). The majority of tumors that did not express GFPT2 also did not express c-Myc (23/25 tumors) (Fig. 1b) (Table 5). Tumors that did not express c-Myc but were positive for GFPT2 expression, showed predominantly weak expression of GFPT2 (weak expression of GFPT2 was observed in 15/16 cases negative for c-Myc and only 1/16 cases negative for c-Myc has strong expression of GFPT2). In addition, expression of c-Myc protein was significantly associated with subtype 2 of LMS (Chi-square test two-sided *p* value = 0.003) (Table 3). Among the 19 cases that expressed c-Myc, 68% (*n* = 13) belonged to subtype 2, 26% (*n* = 5) belonged to subtype 1, and 5% (*n* = 1) belonged to subtype 3 of LMS. In contrast, tumors that did not express c-Myc were classified mostly as subtype 1 and 3 (51% and 26%, respectively). These results show that the expression of c-Myc in LMS is significantly associated with subtype 2 of LMS and with the expression of GFPT2 protein.

**Table 5:** Correlation between cytoplasmic expression of GFPT2 and nuclear expression of c-Myc in 58 LMS cases.

	c-Myc (+)	c-Myc (−)	Two-sided Fisher’s exact test *p* value
GFPT2 (+)	17	16	0.0006
GFPT2 (−)	2	23	

## Discussion

The growth and proliferation of cancer cells is dependent on the reprogramming of cell metabolism. Our findings indicate that the genes involved in the HBP are consistently enriched in primary, recurrent, and metastatic tumors assigned to subtype 2 of LMS in three independent gene expression studies. Importantly, these independent transcriptomic datasets were generated using three distinct technologies (3SEQ, whole transcriptome RNA-Seq, and microarrays), thus our analysis provides a cross-platform validation of the HBP activation in one of the subtypes of LMS. Activation of the genes involved in glucose metabolism utilized for the synthesis of hexosamines is a novel finding in LMS.

The first and rate-limiting step of the HBP is catalyzed by glucose transporter proteins encoded by two highly homologous genes *GFPT1* and *GFPT2*, which convert fructose-6-phosphate to glucosamine-6-phosphate (Fig. 3). In the next steps, glucosamine-6-phosphate is further metabolized until it becomes converted into uridine diphosphate N-acetylglucosamine (UDP-GlcNAc) (Fig. 3). UDP-GlcNAc can be utilized by two principal processes: (1) O-GlcNAcylation, which is a single sugar conjugation, catalyzed by O-GlcNAc transferase (OGT), and (2) O- and N-linked glycosylation leading to complex sugar conjugates on target proteins^21,22^. Increased flux of glucose into HBP leads to altered O-GlcNAcylation and N-/O-glycosylation, which in turn interferes with stability and interactions of key transcription factors, kinases, and cytoplasmic enzymes in many types of cancer^9,10^. These modifications regulate protein functions in several cancer-associated processes, including increased proliferation, invasion, metastasis, and survival of tumor cells, and may be associated with resistance to therapy^11,12^. In the present study we show that LMS tumors with high expression of genes implicated in HBP also show enrichment of multiple genes regulated by c-Myc, a transcription factor and oncoprotein that is known to be stabilized by O-GlcNAcylation. Through the transcriptomic analysis and validation by IHC, we show a correlation between expression of GFPT2 and c-Myc in a subset of LMS. Based on these preliminary findings we hypothesize that the activation of HBP may potentially stabilize c-Myc through O-GlcNAcylation in subtype 2 of LMS. This may lead to altered gene transcription in these tumors, but these results warrant further confirmation. c-Myc is widely recognized as a potent oncoprotein in human tumors. c-Myc heterodimerizes with MAX and regulates gene transcription in cell proliferation, cell differentiation, and programmed cell death^23^. c-Myc is modified by O-linked N-acetylglucosamine (O-GlcNAc) at threonine 58, which is also a known phosphorylation site of this protein^23^. This posttranslational modification is catalyzed by OGT and the gene encoding the OGT enzyme was also significantly overexpressed in subtype 2 of LMS. Stabilization of c-Myc as a consequence of activation of HBP and O-GlcNAc modification has been demonstrated, e.g., in prostate cancer cells^24^. Nevertheless, c-Myc may not be the only target of HBP activation in LMS. In the present study, based on the gene expression profiles of LMS, we attempted to identify possible transcription factors that may be affected by aberrant glycosylation. However, glycosylation may also regulate the function of other proteins relevant for oncogenesis, including protein kinases and oncogenes. In the present study we found overexpression of genes encoding several enzymes of HBP, and the enzymes involved in N- and O-linked glycosylation downstream of this pathway (including OGT and GALE). Thus, we hypothesize that there may be a broader spectrum of oncogenic factors that show aberrant glycosylation in subtype 2 of LMS that warrant further studies.

In this study we report a frequent expression of GFPT2 and c-Myc proteins in subset 2 of LMS (Table 3). We sought to determine whether the genes encoding these proteins are known to be affected in LMS by any genomic aberrations that may contribute to their increased expression on mRNA and protein level. According to the data available from 80 primary LMS included in the TCGA study (accessed on cBioPortal on 12/01/2020), there were no somatic mutations in the *GFPT2* gene, and amplification of *GFPT2* was detected only in 1/80 (1.3%) of these LMS tumors. In addition, the *MYC* gene was not affected by any somatic mutations in these cases either, and was amplified only in 3/80 (4%) of LMS. Therefore, we conclude that the expression or increased activity of GFPT2 or c-Myc in LMS is not regulated by the activating mutations or increased copy number of these genes, and that there are different mechanisms underlying the activation of these proteins in a subset of LMS.

Based on the gene expression analysis, we identified the enrichment of *GFPT2* expression and other genes of HBP in subtype 2 of LMS. Among the 22 cases classified as subtype 2 of LMS in our study, there were 13 uterine tumors and 9 extra-uterine tumors^6^. However, in the survival analysis, we demonstrate that the expression GFPT2 protein is associated with poor clinical outcome in patients with uterine LMS and not in extra-uterine LMS. These results indicate that the activation of HBP in subtype 2 of LMS may have a different effect on the clinical outcome depending on the site of the tumor. This observation warrants further studies in LMS with molecular subtype classification and clinical outcome available.

Given that the future functional studies would confirm the activation of the HBP and stabilization of c-Myc by O-GlcNAcylation in a subset of LMS, these findings may potentially have therapeutic implications in this disease. Targeting O-GlcNAcylation and N-/O-glycosylation in cancer may either directly inhibit cell invasion and tumorigenicity both in vitro and in vivo, or modulate sensitivity to chemo-, radio- and immunotherapy^10,13^. O-GlcNAcylation may be reduced in cancer cells either through small molecule inhibitors of OGT or through inhibitors mimicking UDP-GlcNAc, the substrate of OGT^25,26^. For example, a small molecule inhibitor of OGT, called ST045849, leads to loss of c-Myc activity and cell death in prostate cancer cells when used in combination with alanine amido-transferase inhibitors^27^. Inhibition of OGT has been also demonstrated to synergize with GDC-0941 PI3K inhibitor in multiple cancer cell lines^28^. Inhibition of N-glycosylation affects protein folding and may lead to reduced activity of receptor tyrosine kinases, such as EGFR, ERBB2, IGF1R, and MET^10^. For example, tunicamycin is an inhibitor of N-glycosylation that affects cell invasion, tumor-induced angiogenesis, and sensitivity to chemo- and radiotherapy both in vitro and in vivo in multiple cancer models^10^. Also, selective inhibition of O-glycosylation was demonstrated to induce apoptosis in selected in vitro models of cancer^29^. Targeting tumor-intrinsic hexosamine biosynthesis by direct inhibition of GFPT1/2 by a small molecule glutamine analog (6-diazo-5-oxo-l-norleucine, DON) was recently shown to sensitize pancreatic cancer to anti-PD1 therapy and to result in tumor regression and prolonged survival^13^. This strategy may be also considered in LMS to increase the currently limited efficacy of immunotherapy in this disease. Evaluation of therapeutic strategies targeting O-GlcNAcylation and N-/O-glycosylation in the preclinical models of LMS warrants further studies. A different approach to metabolism-modulating therapy, based on arginine deprivation, has recently showed promising results in preclinical models of LMS^30–32^.

Metabolic reprogramming of tumor cells is often associated with an increased glucose consumption. This can be measured by the SUV_max_ based on the ^18^F-FDG PET images of the tumor. Only a few small published series evaluated metabolic activity of LMS using ^18^F-FDG PET. In a series of 38 primary LMS, Punt et al. demonstrated that SUV_max_ was associated with tumor grade and tumor size, but not with anatomic location^33^. Another series published by Macpherson et al. showed comparable SUV_max_ in 14 uterine and 54 extra-uterine LMS^34^. In a separate study focused only on uterine LMS, an increased SUV_max_ was prognostic of poor survival^35^. In that study, in a univariate Cox model based on 19 patients with uterine LMS, SUV_max_ was the only factor that was significantly correlated with overall survival, as opposed to 8 other variables^35^. These findings correlate with our observation that elevated expression of GFPT2, a mediator of metabolic reprogramming linked with increased glucose uptake, is associated with poor outcome in uterine LMS. In our small imaging cohort we did not have SUV_max_ available from any uterine LMS. Based on a limited number of extra-uterine cases, we propose that overexpression of GFPT2 may be potentially associated with increased glucose uptake in primary LMS. However, this observation needs to be confirmed by further studies in a larger cohort of patients analyzed by ^18^F-FDG PET with corresponding LMS tumor specimens available for molecular studies that would allow testing this hypothesis.

Kusunoki et al. demonstrated an increased metabolic activity as measured by SUV_max_ in uterine LMS compared to benign uterine leiomyomas, and it has been proposed that ^18^F-FDG PET imaging may help in preoperative distinction between these tumors^36,37^. Uterine leiomyomas may indeed have substantially lower metabolic activity also as measured by the GFPT2 protein expression. TMAs that we analyzed in the present study included cores from four uterine leiomyomas, all of which were negative for GFPT2 expression (data not shown).

In summary, we discovered a significant enrichment of genes involved in HBP in subtype 2 of LMS in three independent datasets. The activation of HBP, and more specifically, high expression of GFPT2, is a novel observation in LMS. Through evaluation of several independent cohorts of LMS patients, we demonstrated the prognostic value of GFPT2 expression in primary uterine tumors, we showed the consistency of GFPT2 expression in disease progression and found a possible association between GFPT2 expression and glucose uptake measured by ^18^F-FDG PET in primary LMS. We propose c-Myc as a possible protein stabilized by O-GlcNAcylation as a result of the activated HBP in subtype 2 of LMS. Overall, our findings suggest subtype-specific metabolic reprogramming in LMS and suggest that HBP may be a potential new therapeutic target in these tumors.

## Methods

### Patients

In this study, we analyzed three independent cohorts of LMS patients (Table 1):

1. Stanford LMS subtypes cohort: we used gene expression data obtained from a previously published cohort of 99 LMS (GSE45510)^6^. Seventy of these 99 specimens were previously classified into distinct transcriptomic subtypes (35, 22, and 13 patients classified into subtypes 1, 2, and 3, respectively) based on Consensus Clustering and positive silhouette value. Cores from formalin fixed paraffin embedded (FFPE) tissue specimens from 59/70 of these patients were included on TMA TA-381. We performed the validation of selected findings from this cohort by re-analyzing the previously published LMS datasets^5,7^.
2. Survival analysis cohort: we assembled a new cohort of 327 patients with primary LMS that presented with localized disease and had clinical follow up available. This cohort consisted of cases collected by the Leiomyosarcoma Direct Research Foundation (LMSdr) and deposited in LMS Paraffin Tissue Bank at Stanford University (*n* = 179), cases collected at Stanford (*n* = 91), and cases collected at MD Anderson Cancer Center (*n* = 57). Cores from FFPE tissue specimens from these patients were included on TMAs TA-201 (cases from Stanford), TAs-464-466 (cases collected by LMSdr), and TAs-541-548 (cases from MD Anderson Cancer Center). From 37 patients in this survival analysis cohort, there were multiple tumor specimens available on TMAs (from primary, local recurrence, and/or distant metastatic tumors). These specimens were used to evaluate the consistency of GFPT2 protein expression in primary tumors and relapse.
3. Imaging cohort: we identified 16 patients from whom the archival material from tumors analyzed by ^18^F-FDG-PET imaging was available. For these patients, we examined whole sections of the FFPE tumor or biopsy material by IHC.

The study was approved by the Institutional Review Boards of Stanford University and MD Anderson Cancer Research Center. This study used archival material for which waiver of consent was granted from the Institutional Review Boards.

### Gene expression analysis

GSEA was performed using GSEA Mac App (version 4.0.3)^16^. GSEA was performed using weighted enrichment statistic, 1000 permutations and the time stamp as the seed value. Fourteen gene sets representing carbohydrate metabolism pathways that are curated in KEGG database (accessed on 03/05/2020) and C3 TFT collection curated in MSigDB were used for GSEA^15,16^. A FDR <0.25 was used to identify significantly enriched gene sets, as recommended in the GSEA manual. GSEA with the same settings was also applied to the datasets of LMS cases published by Chudasama et al., Beck et al., and by TCGA^5,7,8^. The enrichment plot in Fig. 1a was generated using replotGSEA function from the Rtoolbox package in R (version 3.6.1) (package accessed from https://github.com/PeeperLab/Rtoolbox on 12/07/2020).

Correlation between *GFPT2* gene expression levels and GFPT2 protein expression was performed using mRNA read counts normalized with variance stabilizing transformation from DESeq2 package (version 1.24.0)^38^. Multiclass differential expression analysis was performed in R (version 3.6.1) with the samr package (version 3.0)^39^. SAMseq function from samr package was applied to raw read counts generated with 3′-end sequencing from 70 LMS tumors previously assigned to three molecular subtypes (GSE45510)^6^. Genes overexpressed in each subtype of LMS at contrast >2 compared to the other groups, and FDR < 0.05 were identified in each group (with default settings and seed value for SAMseq = 1,234,567).

### Immunohistochemistry

Histopathological examination was performed on TMAs constructed from FFPE tissues (for the Stanford LMS subtypes cohort and the survival cohort), and on the whole sections of the tumor or biopsy material (for the imaging cohort). Sections (5 μm) were used for routine hematoxylin and eosin staining, and for IHC staining by avidin-biotin-peroxidase complex method. Expression of GFPT2 and c-Myc was evaluated using the following antibodies: for GFPT2, we used the recombinant rabbit monoclonal antibody [EPR19095] diluted 1:50 (catalog number ab190966, Abcam, Cambridge, UK) with citrate buffer pH = 6 for antigen retrieval; for c-Myc, we used the recombinant rabbit monoclonal antibody [Y69] diluted 1:100 (catalog number ab32072, Abcam, Cambridge, UK) with the BOND Epitope Retrieval solution 2 pH = 9 (catalog number AR9640, Leica) for antigen retrieval. Appropriate positive and negative controls were run in parallel. Stained TMA slides were scanned into the Stanford Tissue Microarray Database (TMAD). Staining intensity was scored qualitatively and classified into three categories for cytoplasmic GFPT2 expression and nuclear c-Myc expression: negative, weak, and strong.

Among the 202 cases with duplicate cores available on TMAs stained for GFPT2, we observed uniform expression of this protein in 190 (94%) of cases. Among the 12 cases with different scores between duplicate cores, 9 cases showed strong expression of GFPT2 in one core and weak expression in the other core, and 3 cases showed weak expression in one core and did not express GFPT2 in the other core. All discordant cases belonged to the survival cohort. For the purpose of the survival analysis, the three tumors with GFPT2 expression observed in only one of the cores were classified as positive for GFPT2 expression, and LMS cases with weak and strong expression of GFPT2 were grouped together as the “GFPT2 positive” cases. Images of stained TMAs are available to the public on TMAD website at https://tma.im (for TA-201, TA-381, TAs-464-466, and TAs-541-548).

### Evaluation of glucose uptake by ^18^F-FDG-PET

The images were acquired using GE model 600 PET-CT scanner at Stanford University Medical Center. Maximum standard uptake values (SUV_max_) were extracted from retrospective routine diagnostic reports from ^18^F-FDG-PET imaging. SUV_max_ values were defined as the ratio of ^18^F-FDG uptake per unit volume of the region of interest to the uptake per unit volume of the whole body. For descriptive purposes, the SUV_max_ of metabolically active tissues was reported in g/ml. Information from diagnostic surgical pathology reports was used to ensure that GFPT2 protein expression was evaluated in the tumor specimen matching with the imaging report.

### Statistical methods

For contingency analyses, we combined the groups with weak and strong expression of GFPT2 protein in order to meet the requirements of these statistical tests (minimum of one sample per group and at least 20% of the expected values are greater than 5). *p* values have been computed using Fisher’s exact test for 2 × 2 contingency tables and using Chi-square test for contingency tables larger than 2 × 2, using GraphPad Prism software version 8.3.

Comparison of mean mRNA expression values of GFPT2 between the tumors with strong, weak and no expression of GFPT2 protein was performed by ordinary one-way ANOVA test using GraphPad Prism software version 8.3.

Survival analysis was performed using GraphPad Prism software version 8.3. Risk groups were defined as negative for GFPT2 expression and positive for GFPT2 expression (either weak or strong expression). The Mantel–Cox log-rank test was used to calculate the *p* value, hazard ratio, and 95% confidence interval of the ratio. Kaplan–Meier curves in Fig. 1d, e and in Supplementary Fig. 1 were plotted in R using survival and survminer packages in R (version 3.6.1).

### Reporting summary

Further information on research design is available in the Nature Research Reporting Summary linked to this article.

## Supplementary information

Supplementary Information

Reporting Summary

## Data Availability

Four transcriptomic datasets used in this study are publicly available in the following repositories: gene expression microarray data from 51 LMS published by Beck et al. is available in the Gene Expression Omnibus under accession number GSE17555^5^; 3SEQ gene expression data from 99 LMS published by Guo et al. is available in the Gene Expression Omnibus under accession number GSE45510^6^; whole transcriptome RNA-Seq data from 37 LMS published by Chudasama et al. is available in the European Genomephenome Archive under accession number EGAS00001002437^7^, and whole transcriptome RNA-Seq data from 80 LMS included in the TCGA SARC project is available in the Genomic Data Commons (https://gdc.cancer.gov)^8^. Images of stained tissue microarrays are available to the public on TMAD website at https://tma.im (for TA-201, TA-381, TAs-464-466, and TAs-541-548).

## Code availability

R packages used in this study (R version 3.6.1): survival (version 3.2-7), survminer (version 0.4.8), samr (version 3.0), DESeq2 (version 1.24.0), Rtoolbox (version 1.4). Software used in this study: GraphPad Prism software version 8.3, GSEA version 4.0.3.
