# Supplementary material for: Prognostic relevance of the hexosamine biosynthesis pathway activation in leiomyosarcoma
Source: NPJ Genom Med. 2021 May 3;6:30. doi: 10.1038/s41525-021-00193-w (PMC8093268; doi:10.1038/s41525-021-00193-w)
Supplement: Supplementary file 1 — Supplementary Information [file 41525_2021_193_MOESM1_ESM.pdf]

Supplementary Figure 1
Tolwani et al.

a

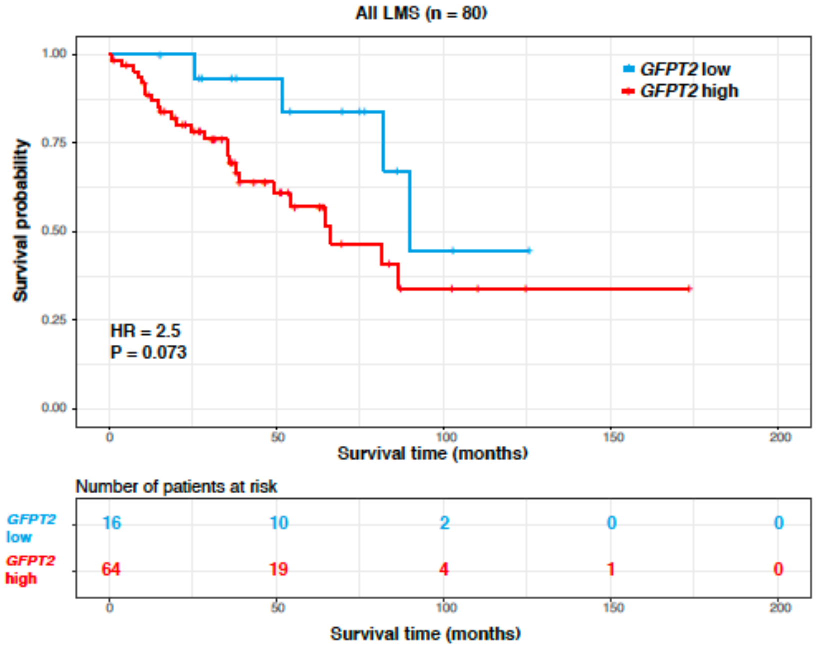

b

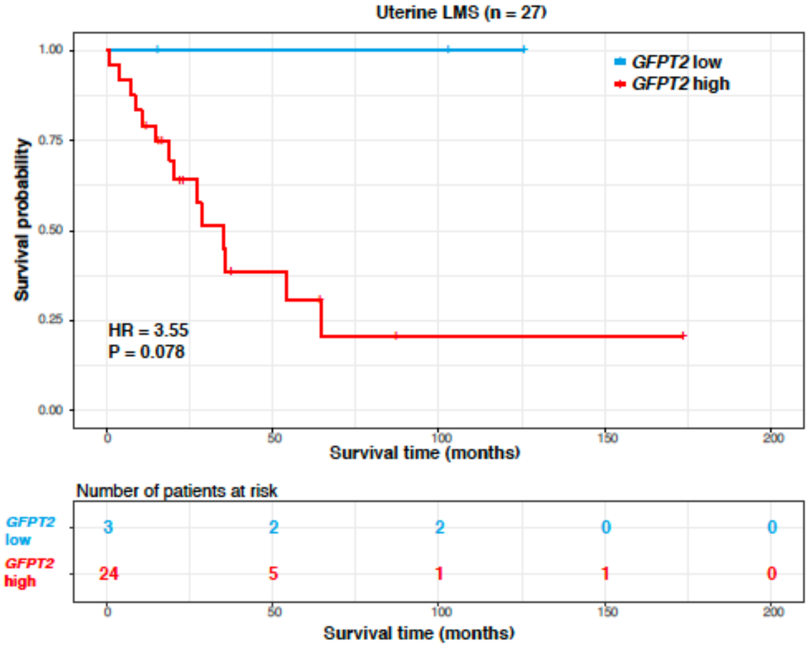

Correlation between the overall survival and *GFPT2* mRNA expression in A) 80 patients with uterine and extra-uterine LMS, and B) 27 patients with uterine LMS analyzed separately, included in the TCGA study. Tumors with “high” expression of *GFPT2* had RSEM expression values above 20th percentile (red line) as opposed to tumors with “low” expression with *GFPT2* RSEM values below 20th percentile (blue line). Censored observations are indicated on the Kaplan-Meier curves with a cross. P values were calculated using log-rank (Mantel-Cox) test, and hazard ratios were calculated using log-rank method. (HR - hazard ratio)

Supplementary Table 1
Gene Set Enrichment for KEGG metabolic pathways in the series of 52 primary and 18 recurrent/metastatic LMS from Guo et al. dataset

Group of specimens	Gene set	Enrichment Score (ES)	Normalized Enrichment Score (NES)	FDR q-value	Number of genes in core enrichment
Primary - Subtype 1	KEGG_INOSITOL_PHOSPHATE_METABOLISM	0.518216	1.636454	0.10221852	27/54
Primary - Subtype 2	KEGG_CITRATE_CYCLE_TCA_CYCLE	0.6287474	1.7153925	0.03191925	15/30
Primary - Subtype 2	KEGG_AMINO_SUGAR_AND_NUCLEOTIDE_SUGAR_METABOLISM	0.5235722	1.4542532	0.23037258	27/43
Primary - Subtype 2	KEGG_GALACTOSE_METABOLISM	0.49655837	1.4002521	0.21491155	15/26
Primary - Subtype 2	KEGG_PENTOSE_PHOSPHATE_PATHWAY	0.44638783	1.3368784	0.2242881	13/25
Primary - Subtype 3	KEGG_ASCORBATE_AND_ALDARATE_METABOLISM	0.50474757	1.5214751	0.05260692	10/25
Primary - Subtype 3	KEGG_PENTOSE_AND_GLUCURONATE_INTERCONVERSIONS	0.33969408	1.141602	0.22769222	9/27
Recurrent/metastatic - Subtype 1	No gene sets enriched				
Recurrent/metastatic - Subtype 2	KEGG_CITRATE_CYCLE_TCA_CYCLE	0.5901765	1.6920705	0.022774149	16/30
Recurrent/metastatic - Subtype 2	KEGG_AMINO_SUGAR_AND_NUCLEOTIDE_SUGAR_METABOLISM	0.3886516	1.3150951	0.18733697	13/43
Recurrent/metastatic - Subtype 3	Not applicable (only 1 sample)				

Supplementary Table 2
Gene Set Enrichment for KEGG metabolic pathways in the series of 37 primary and recurrent/metastatic LMS published by Chudasama et al.

Group of specimens	Gene set	Enrichment Score (ES)	Normalized Enrichment Score (NES)	FDR q-value	Number of genes in core enrichment
Subgroup 1	KEGG_GLYCOLYSIS_GLUCONEOGENESIS	0.71754915	2.1650805	9.68E-04	15/62
Subgroup 1	KEGG_STARCH_AND_SUCROSE_METABOLISM	0.8472251	2.065428	4.84E-04	19/52
Subgroup 1	KEGG_INOSITOL_PHOSPHATE_METABOLISM	0.49818185	2.060278	3.23E-04	13/54
Subgroup 1	KEGG_FRUCTOSE_AND_MANNOSE_METABOLISM	0.66460764	2.0375528	6.66E-04	7/33
Subgroup 1	KEGG_PYRUVATE_METABOLISM	0.5987818	1.9242563	0.003207706	6/40
Subgroup 1	KEGG_GLYOXYLATE_AND_DICARBOXYLATE_METABOLISM	0.77172524	1.8644878	0.006085359	4/16
Subgroup 1	KEGG_CITRATE_CYCLE_TCA_CYCLE	0.6098534	1.8185745	0.008918148	4/31
Subgroup 1	KEGG_PENTOSE_AND_GLUCURONATE_INTERCONVERSIONS	0.9058163	1.7902175	0.010548012	15/27
Subgroup 1	KEGG_PROPANOATE_METABOLISM	0.5510952	1.7733748	0.011145111	16/32
Subgroup 1	KEGG_ASCORBATE_AND_ALDARATE_METABOLISM	0.915854	1.7672243	0.013868181	15/25
Subgroup 1	KEGG_PENTOSE_PHOSPHATE_PATHWAY	0.545891	1.7244595	0.018557386	3/25
Subgroup 1	KEGG_BUTANOATE_METABOLISM	0.6793269	1.7118028	0.018556379	10/34
Subgroup 1	KEGG_GALACTOSE_METABOLISM	0.40936092	1.3100709	0.15560018	8/26
Subgroup 2	KEGG_AMINO_SUGAR_AND_NUCLEOTIDE_SUGAR_METABOLISM	0.46511623	1.6197191	0.19426829	23/43
Subgroup 3	No enrichment				

Supplementary Table 3

Gene Set Enrichment for KEGG metabolic pathways in the series of 51 primary and recurrent/metastatic LMS published by Beck et al.

Group of specimens	Gene set	Enrichment Score (ES)	Normalized Enrichment Score (NES)	FDR q-value	Number of genes in core enrichment
Subtype 1	KEGG_INOSITOL_PHOSPHATE_METABOLISM	0.39637566	1.494577	0.2245795	16/47
Subtype 1	KEGG_BUTANOATE_METABOLISM	0.406455	1.3890061	0.20791367	8/27
Subtype 2	KEGG_AMINO_SUGAR_AND_NUCLEOTIDE_SUGAR_METABOLISM	0.25845802	0.9118567	0.5347829	10/36
Subtype 3	KEGG_PENTOSE_PHOSPHATE_PATHWAY	0.49971318	1.5211684	0.22440048	7/22
Subtype 3	KEGG_GLYCOLYSIS_GLUCONEOGENESIS	0.42340752	1.4601891	0.19360389	22/48
Subtype 3	KEGG_GALACTOSE_METABOLISM	0.49743298	1.3778338	0.2052903	11/20
Subtype 3	KEGG_FRUCTOSE_AND_MANNOSE_METABOLISM	0.43824795	1.3426803	0.18101211	18/29

Supplementary Table 4
Core genes from the amino sugar and nucleotide sugar metabolism gene set enriched in subtype 2 LMS within the 52 primary tumors from Guo et al. dataset

Gene	Rank in gene list	Rank metric score	Running ES	Core enrichment
UGP2	48	0.671091735	0.05675121	Yes
GALK1	319	0.541462481	0.092075944	Yes
UAP1	341	0.536036193	0.1381907	Yes
PGM3	724	0.453770816	0.1607483	Yes
HEXB	763	0.448375314	0.19839709	Yes
GALE	823	0.438832998	0.23425785	Yes
GMPPB	913	0.427784681	0.26779115	Yes
AMDHD2	1197	0.394686639	0.2896405	Yes
PGM2	1220	0.391334385	0.3230049	Yes
GNPDA1	1328	0.38171339	0.35167864	Yes
PMM1	1636	0.354264319	0.3688929	Yes
GMDS	2371	0.301830947	0.36218303	Yes
GMPPA	2567	0.291162103	0.37892446	Yes
NANS	2614	0.288255244	0.40215242	Yes
GFPT2	2710	0.282816112	0.42268574	Yes
PMM2	2752	0.280481339	0.4454574	Yes
NAGK	2997	0.268339455	0.45797783	Yes
HEXA	3489	0.242583618	0.45706093	Yes
UGDH	3728	0.231761441	0.46664125	Yes
HK1	3833	0.227867857	0.4819428	Yes
CYB5R3	3888	0.226029456	0.49934527	Yes
CYB5R1	4584	0.19730626	0.48522255	Yes
TSTA3	4759	0.19024007	0.49405304	Yes
MPI	4833	0.186565891	0.50713086	Yes
HK2	5288	0.166468501	0.501205	Yes
HK3	5329	0.164785773	0.5138636	Yes
GPI	5428	0.161076039	0.5235722	Yes

Supplementary Table 5

Core genes from the amino sugar and nucleotide sugar metabolism gene set enriched in subtype 2 LMS within 18 recurrent and metastatic tumors from Guo et al. dataset

Gene	Rank in gene list	Rank metric score	Running ES	Core enrichment
GFPT2	226	0.705980062	0.059595436	Yes
HEXB	416	0.617715657	0.11213567	Yes
UAP1	434	0.60977596	0.17167313	Yes
NANS	1039	0.472532004	0.19107732	Yes
PGM3	1084	0.465644062	0.23513852	Yes
HK1	1670	0.393057466	0.2475424	Yes
RENBP	1756	0.384537846	0.28172708	Yes
GNPDA2	2300	0.345108986	0.29128924	Yes
FPGT	2405	0.335926503	0.3198066	Yes
UGP2	2686	0.311540693	0.33794877	Yes
UGDH	2963	0.291161835	0.35425645	Yes
GALK1	3173	0.276280075	0.3721239	Yes
UXS1	3382	0.26227656	0.3886516	Yes

Supplementary Table 6

Core genes from the amino sugar and nucleotide sugar metabolism gene set enriched in subgroup 2 LMS in Chudasama et al. dataset

Gene	Rank in gene list	Rank metric score	Running ES	Core enrichment
GFPT2	544	0.620428085	0.08987255	Yes
HK3	816	0.554523826	0.17420761	Yes
HK2	1758	0.426317751	0.22539608	Yes
NPL	3517	0.31739229	0.24380736	Yes
RENBP	5890	0.214625806	0.23421583	Yes
UGDH	6411	0.197797835	0.2564119	Yes
NANS	7162	0.177150935	0.27099535	Yes
NAGK	7360	0.171843007	0.2950248	Yes
UXS1	7366	0.171727166	0.3226123	Yes
CHIT1	7451	0.169312105	0.3483388	Yes
CYB5R1	8318	0.147597328	0.35599762	Yes
HEXA	9305	0.12671493	0.35805497	Yes
GMPPB	9338	0.126150429	0.37779298	Yes
GALE	9604	0.121560007	0.3924506	Yes
PGM3	9868	0.117060401	0.4064202	Yes
PMM2	10821	0.100434788	0.40487483	Yes
GNPNAT1	10881	0.099256486	0.41977486	Yes
GALK1	11590	0.087891623	0.42075303	Yes
HK1	11628	0.087251201	0.43412775	Yes
HEXB	11754	0.085576698	0.44559324	Yes
GMDS	11798	0.084828116	0.4584656	Yes
PMM1	12219	0.078387082	0.4632768	Yes
PGM1	12733	0.070698731	0.46511623	Yes

Supplementary Table 7

Core genes from the amino sugar and nucleotide sugar metabolism gene set enriched in subgroup 2 LMS in Beck et al. dataset

Gene	Rank in gene list	Rank metric score	Running ES	Core enrichment
GFPT2	109	0.770824492	0.061938025	Yes
UAP1	502	0.582042933	0.087159894	Yes
UXS1	557	0.569466174	0.13476376	Yes
PGM3	721	0.529480696	0.17117763	Yes
UGP2	1062	0.462604672	0.18924509	Yes
NPL	1111	0.455178082	0.22695862	Yes
UGDH	1268	0.432703465	0.25513104	Yes
GFPT1	1841	0.362207562	0.24800217	Yes
GNPDA2	2371	0.312324882	0.23936605	Yes
GALK2	2488	0.301166922	0.25845802	Yes

Supplementary Table 8

Significant differentially expressed genes involved in amino sugar and nucleotide sugar metabolism in subtype 2 of LMS.

	Subtype 1	Subtype 2	Subtype 3	FDR
GFPT2	-1.199	2.835	-1.843	0.00112
HEXB	-2.252	3.675	-1.491	0.00005
UAP1	-1.586	3.929	-2.651	0
NANS	-0.27	2.632	-2.795	0.00031
PGM3	-3.55	3.024	0.954	0.00007
HK1	1.758	2.231	-4.924	0
UGP2	-4.294	4.347	0.332	0
UGDH	-2.046	2.361	-0.187	0.00887
GALK1	-2.262	5.701	-3.897	0
GALE	0.859	2.891	-4.555	0
GMPPB	0.804	3.236	-4.896	0
AMDHD2	-0.985	3.88	-3.366	0
GNPDA1	-1.142	2.455	-1.463	0.00608
PMM1	-0.719	3.857	-3.681	0
GMDS	-1.981	2.437	-0.362	0.00758
GMPPA	1.297	2.809	-5.021	0
NAGK	1.63	2.571	-5.164	0
HEXA	1.818	2.39	-5.191	0
CYB5R1	0.263	2	-2.726	0.00163

Supplementary Table 9

Gene Set Enrichment for C3:TFT gene sets in the series of 52 primary and 18 recurrent/metastatic LMS from Guo et al. dataset

Gene set	Enrichment Score (ES)	Normalized Enrichment Score (NES)	FDR q-value
GGAANCGGAANY_UNKNOWN	0.5171128	1.7678894	0.088920265
GCGNNANTTCC_UNKNOWN	0.460976	1.7304499	0.10054375
ELK1_02	0.47456264	1.806565	0.106319636
E2F_Q4_01	0.39555117	1.5966474	0.15597008
KCCGNSWTTT_UNKNOWN	0.4282569	1.575337	0.16092171
CGGAARNGGCNG_UNKNOWN	0.45907366	1.5831676	0.16120026
GCCATNTTG_YY1_Q6	0.39365497	1.5653192	0.1628356
GGCNNMSMYNTTG_UNKNOWN	0.45960563	1.6014062	0.16457036
CCGNMNNTNACG_UNKNOWN	0.475897	1.6636243	0.16696323
TAANNYSGCG_UNKNOWN	0.43399274	1.6076598	0.17821214
CETS1P54_01	0.3809466	1.5466063	0.18614425
GABP_B	0.4102795	1.6125504	0.19750248
AHR_01	0.3796473	1.4443156	0.20349455
NRF2_01	0.4003257	1.518131	0.203765
TTCNRGNNNNTTC_HSF_Q6	0.36896193	1.4470041	0.20479688
USF_Q6	0.37469143	1.5236074	0.2055949
CREB_Q2	0.36499065	1.4793737	0.20775147
ETS_Q4	0.35053495	1.4355286	0.20822106
E2F1_Q4_01	0.35407808	1.452427	0.20871796
HIF1_Q3	0.35894614	1.4313822	0.21054459
KTGGYRSGAA_UNKNOWN	0.39869848	1.4475977	0.2105863
TEL2_Q6	0.36507544	1.4278325	0.21070917
CGTSACG_PAX3_B	0.38810217	1.4868724	0.21135749
NFMUE1_Q6	0.3910095	1.5076747	0.21244287
CREB_02	0.34726492	1.4356354	0.21428432
ACAWNRNSRCGG_UNKNOWN	0.41271374	1.4532313	0.21481234
CREB_01	0.35434204	1.4803989	0.21498841
E2F_03	0.35429913	1.4223725	0.21577324
GGCNRNWCTTYS_UNKNOWN	0.3987143	1.5255331	0.21589322
CREB_Q4_01	0.36074257	1.4610265	0.2158982
CRGAARNNNNCGA_UNKNOWN	0.43202904	1.4894805	0.2166324
TCCCRNNRTGC_UNKNOWN	0.35750934	1.456252	0.21681659
CREB_Q4	0.3703125	1.4992748	0.21853259
USF_01	0.3623316	1.4673728	0.22067526
TTGCWCAAY_CEBPB_02	0.41832086	1.4929339	0.22128692
NERF_Q2	0.36710247	1.46253	0.22173208
SREBP1_01	0.36354744	1.4114057	0.22485696
CYTAGCAAY_UNKNOWN	0.32698065	1.4128282	0.22749041
ARNT_02	0.41141418	1.6147041	0.23114404
MYCMAX_03	0.3380727	1.3931289	0.23364893
E2F_Q3_01	0.3406964	1.3971788	0.23566628
HIF1_Q5	0.34951794	1.3989012	0.23794852
GKCGCNNNNNNNTGAYG_UNKNOW	0.4358071	1.3935615	0.23817877
TMTCGCGANR_UNKNOWN	0.38382265	1.3989464	0.24377133

Supplementary Table 10
Core genes from the "MYCMAX_03" gene set enriched in 22 LMS cases assigned to subtype 2 in Guo et al. dataset

Gene	Rank in gene list	Rank metric score	Running ES	Core enrichment
COPZ1	1	0.847219586	0.015194092	Yes
TRIB1	14	0.710183024	0.027420798	Yes
CEBPB	22	0.693414748	0.039574232	Yes
TXNDC12	36	0.667060733	0.050979584	Yes
RPL13A	61	0.64029187	0.061401047	Yes
HMOX1	188	0.562441528	0.06576375	Yes
ANKRD17	202	0.555335343	0.07515939	Yes
LAMP1	248	0.534894824	0.0827259	Yes
ZCCHC7	343	0.506237626	0.08753906	Yes
ANKHD1	377	0.498912543	0.09500636	Yes
CD164	465	0.479276121	0.09965424	Yes
C16orf72	515	0.467540681	0.1058265	Yes
PABPC1	576	0.456095099	0.11129049	Yes
EIF3A	584	0.455014765	0.11915559	Yes
UCHL1	617	0.448660076	0.12576462	Yes
GNAS	633	0.445724487	0.13309725	Yes
HNRNPA1	652	0.442564189	0.140236	Yes
GJA1	679	0.439183623	0.1469486	Yes
PPCS	680	0.439098626	0.1548471	Yes
NEUROD2	792	0.424532682	0.15741415	Yes
TOM1	816	0.419980049	0.16391833	Yes
C6orf211	873	0.411193132	0.16875732	Yes
PRDM4	1036	0.393106073	0.16842988	Yes
H3F3A	1053	0.390724003	0.17472748	Yes
C1orf43	1137	0.381754667	0.17780383	Yes
ARPC5	1144	0.380991369	0.18438308	Yes
DCTN4	1202	0.374928206	0.18852405	Yes
PRPS1	1231	0.371740639	0.19393215	Yes
SLC38A2	1241	0.370342523	0.20018283	Yes
ODC1	1347	0.360681802	0.20187536	Yes
SPNS1	1359	0.359653294	0.20784241	Yes
MANF	1397	0.357007802	0.21257445	Yes
NOP58	1466	0.350492179	0.21577351	Yes
AFF4	1476	0.349137098	0.22164275	Yes
SEZ6L2	1492	0.348127365	0.22721979	Yes
B3GNT9	1619	0.339259952	0.22756791	Yes
RAB3IL1	1645	0.33733055	0.23249404	Yes
PICALM	1662	0.336531103	0.23781683	Yes
RBM15B	1669	0.336039126	0.24358746	Yes
ADSS	1736	0.329921395	0.24650784	Yes
SEC23IP	1816	0.323068529	0.24871121	Yes
OLFM2	1917	0.316675782	0.24984053	Yes
RPS28	1940	0.315381318	0.25450885	Yes
KBTBD2	2036	0.309374869	0.2557352	Yes
SLC38A5	2075	0.306852281	0.25951937	Yes
GABARAP	2100	0.305502057	0.26391864	Yes
PPM1A	2104	0.305266052	0.26927274	Yes
CTSA	2141	0.302986383	0.2730787	Yes
REXO2	2170	0.300949544	0.2772134	Yes
PBRM1	2211	0.297825307	0.28074387	Yes
FKBP11	2225	0.297111571	0.2854946	Yes
DNMT3A	2277	0.294192374	0.2884573	Yes
PABPC4	2323	0.291431218	0.2916444	Yes
CLIP2	2434	0.285056561	0.29174826	Yes
TGFB2	2503	0.281237215	0.29370153	Yes
HMHA1	2505	0.281037122	0.29871115	Yes
RNF219	2541	0.27905336	0.3021323	Yes
SMC3	2599	0.276007771	0.3044939	Yes
NEUROD1	2822	0.264030427	0.29910442	Yes
PPRC1	2901	0.259740829	0.30021435	Yes
EIF4B	2930	0.258094966	0.30357817	Yes
FAM192A	3028	0.25418058	0.30372036	Yes
FMR1	3088	0.250129998	0.30552512	Yes
PTMA	3108	0.248784348	0.30913252	Yes
AP1S2	3225	0.243603349	0.30821666	Yes
VPS16	3256	0.241906241	0.31119794	Yes
RNF146	3276	0.241130292	0.31466767	Yes
VPS37B	3335	0.238730535	0.31631306	Yes
ZBTB8OS	3365	0.237401307	0.319259	Yes
BAX	3542	0.229204699	0.31534392	Yes
MAT2A	3608	0.226052403	0.31644154	Yes
HOXA7	3763	0.218251407	0.3133342	Yes
IRF9	3854	0.214731663	0.31308645	Yes
KRTCAP2	4031	0.207053915	0.30877292	Yes
HEXA	4061	0.205964729	0.31115338	Yes
STMN1	4218	0.199229315	0.3076125	Yes
FBXL19	4233	0.198748678	0.31054822	Yes
MRPL27	4234	0.198705345	0.31412253	Yes
TADA1	4295	0.1964982	0.3149169	Yes
KIAA0664	4310	0.195874259	0.3178009	Yes
LIG3	4398	0.192326099	0.31728712	Yes
SNX5	4479	0.189394325	0.3170403	Yes
HNRNPD	4502	0.188596115	0.31942803	Yes
SASH3	4516	0.188041702	0.32221678	Yes
ASPHD1	4535	0.187083527	0.32476	Yes
UTP14A	4540	0.186738923	0.32793635	Yes
HOXA1	4653	0.182119638	0.32609722	Yes
SLC17A9	4747	0.178082123	0.32505322	Yes
PTGES2	4769	0.177221701	0.32728198	Yes
AMMECR1L	4857	0.173917353	0.3264371	Yes
NDUFA7	5113	0.16401276	0.31774136	Yes
DYM	5127	0.163358167	0.32008615	Yes
TOPORS	5237	0.159588486	0.31797874	Yes
UBE2B	5286	0.157748565	0.31862414	Yes
XPO1	5319	0.15651536	0.31997806	Yes
RLF	5335	0.155988768	0.32209894	Yes
UBXN6	5412	0.1531661	0.32138312	Yes
COMMD3	5423	0.1528043	0.32367507	Yes
PFDN2	5450	0.151985332	0.32522154	Yes
PHF20L1	5491	0.150454938	0.3261011	Yes
KDM6A	5587	0.147131875	0.324409	Yes
ILF3	5598	0.146667376	0.32659057	Yes
SNCAIP	5638	0.145454869	0.32742584	Yes
C10orf46	5750	0.141278386	0.32489774	Yes
RGL1	5854	0.136745811	0.32265347	Yes
CHD4	5859	0.136583939	0.32492766	Yes
ATP6V1C1	5886	0.135676488	0.32618076	Yes
SYNCRIP	5892	0.135567918	0.32839102	Yes
SIGMAR1	5923	0.134597301	0.32944202	Yes
EIF4G1	5968	0.132586122	0.32981747	Yes
RPA1	5979	0.132270053	0.33174005	Yes
TFAP4	6000	0.131498754	0.33319202	Yes
RELB	6005	0.131187499	0.33536914	Yes
NEURL2	6008	0.131107345	0.33763617	Yes
NPM1	6069	0.128750727	0.3372119	Yes
ATF4	6108	0.127616167	0.33777198	Yes
TRIM37	6152	0.125892669	0.3380727	Yes
