# Supplementary material for: Prognostic relevance of the hexosamine biosynthesis pathway activation in leiomyosarcoma
Source: NPJ Genom Med. 2021 May 3;6:30. doi: 10.1038/s41525-021-00193-w (PMC8093268; doi:10.1038/s41525-021-00193-w)
Supplement: Supplementary file 2 — Reporting Summary [file 41525_2021_193_MOESM2_ESM.pdf]

nature research

Corresponding author(s): Joanna Przybyl

Last updated by author(s): Dec 10, 2020

Reporting Summary

Nature Research wishes to improve the reproducibility of the work that we publish. This form provides structure for consistency and transparency in reporting. For further information on Nature Research policies, see our Editorial Policies and the Editorial Policy Checklist.

Statistics

For all statistical analyses, confirm that the following items are present in the figure legend, table legend, main text, or Methods section.

n/a	Confirmed	
☐	☒	The exact sample size (*n*) for each experimental group/condition, given as a discrete number and unit of measurement
☐	☒	A statement on whether measurements were taken from distinct samples or whether the same sample was measured repeatedly
☐	☒	The statistical test(s) used AND whether they are one- or two-sided<br>*Only common tests should be described solely by name; describe more complex techniques in the Methods section.*
☒	☐	A description of all covariates tested
☒	☐	A description of any assumptions or corrections, such as tests of normality and adjustment for multiple comparisons
☐	☒	A full description of the statistical parameters including central tendency (e.g. means) or other basic estimates (e.g. regression coefficient) AND variation (e.g. standard deviation) or associated estimates of uncertainty (e.g. confidence intervals)
☐	☒	For null hypothesis testing, the test statistic (e.g. *F*, *t*, *r*) with confidence intervals, effect sizes, degrees of freedom and *P* value noted<br>*Give P values as exact values whenever suitable.*
☒	☐	For Bayesian analysis, information on the choice of priors and Markov chain Monte Carlo settings
☒	☐	For hierarchical and complex designs, identification of the appropriate level for tests and full reporting of outcomes
☒	☐	Estimates of effect sizes (e.g. Cohen's *d*, Pearson's *r*), indicating how they were calculated

Our web collection on statistics for biologists contains articles on many of the points above.

Software and code

Policy information about availability of computer code

Data collection	Microsoft Excel
Data analysis	GraphPad Prism software version 8.3, R (version 3.6.1), GSEA Mac App (version 4.0.3)

For manuscripts utilizing custom algorithms or software that are central to the research but not yet described in published literature, software must be made available to editors and reviewers. We strongly encourage code deposition in a community repository (e.g. GitHub). See the Nature Research guidelines for submitting code & software for further information.

Data

Policy information about availability of data

All manuscripts must include a data availability statement. This statement should provide the following information, where applicable:

- Accession codes, unique identifiers, or web links for publicly available datasets
- A list of figures that have associated raw data
- A description of any restrictions on data availability

Four transcriptomic datasets used in this study are publicly available in the following repositories: gene expression microarray data from 51 LMS published by Beck et al. is available in the Gene Expression Omnibus under accession number GSE17555 (5); 3SEQ gene expression data from 99 LMS published by Guo et al. is available in the Gene Expression Omnibus under accession number GSE45510 (6); whole transcriptome RNA-Seq data from 37 LMS published by Chudasama et al. is available in the European Genomephenome Archive under accession number EGAS00001002437 (7) and whole transcriptome RNA-Seq data from 80 LMS included in the TCGA SARC project is available in the Genomic Data Commons (https://gdc.cancer.gov) (8).

Field-specific reporting

Please select the one below that is the best fit for your research. If you are not sure, read the appropriate sections before making your selection.

☒ Life sciences ☐ Behavioural & social sciences ☐ Ecological, evolutionary & environmental sciences

For a reference copy of the document with all sections, see nature.com/documents/nr-reporting-summary-flat.pdf

Life sciences study design

All studies must disclose on these points even when the disclosure is negative.

Sample size	We used previously published gene expression datasets, therefore the sample size calculation is not applicable. For the survival analyses and imaging analysis, we accessed the available tumor specimens collected by LMSdr, Stanford Cancer Center and MD Anderson Cancer Center.
Data exclusions	No data exclusion has been made
Replication	For 202 tumor specimens we analyzed duplicate cores to evaluate the concordance of protein expression between different regions of the tumor.
Randomization	Our analyses did not require any randomization
Blinding	Our analyses did not require any blinding

Reporting for specific materials, systems and methods

We require information from authors about some types of materials, experimental systems and methods used in many studies. Here, indicate whether each material, system or method listed is relevant to your study. If you are not sure if a list item applies to your research, read the appropriate section before selecting a response.

Materials & experimental systems

n/a	Involved in the study	
☐	☒	Antibodies
☒	☐	Eukaryotic cell lines
☒	☐	Palaeontology and archaeology
☒	☐	Animals and other organisms
☒	☐	Human research participants
☒	☐	Clinical data
☒	☐	Dual use research of concern

Methods

n/a	Involved in the study	
☒	☐	ChIP-seq
☒	☐	Flow cytometry
☒	☐	MRI-based neuroimaging

Antibodies

Antibodies used	For GFPT2, we used the recombinant rabbit monoclonal antibody [EPR19095] diluted 1:50 (catalog number 190966, Abcam, Cambridge, UK). For c-Myc, we used the recombinant rabbit monoclonal antibody [Y69] diluted 1:100 (catalog number ab32072, Abcam, Cambridge, UK).
Validation	https://www.abcam.com/gfpt2-antibody-epr19095-ab190966.html<br>https://www.abcam.com/c-myc-antibody-y69-ab32072.html
